# Next generation sequencing of chromosomal rearrangements in patients with split-hand/split-foot malformation provides evidence for *DYNC1I1* exonic enhancers of *DLX5/6* expression in humans

**DOI:** 10.1136/jmedgenet-2013-102142

**Published:** 2014-01-23

**Authors:** Hana Lango Allen, Richard Caswell, Weijia Xie, Xiao Xu, Christopher Wragg, Peter D Turnpenny, Claire L S Turner, Michael N Weedon, Sian Ellard

**Affiliations:** 1 Institute of Biomedical and Clinical Science, University of Exeter Medical School, Exeter, UK; 2 Bristol Genetics Laboratory, North Bristol NHS Trust, Bristol, UK; 3 Department of Clinical Genetics, Royal Devon & Exeter NHS Foundation Trust, Exeter, UK

**Keywords:** Clinical Genetics, Copy-Number, Genetics, Chromosomal

## Abstract

**Objective:**

Split-hand/foot malformation type 1 is an autosomal dominant condition with reduced penetrance and variable expression. We report three individuals from two families with split-hand/split-foot malformation (SHFM) in whom next generation sequencing was performed to investigate the cause of their phenotype.

**Methods and results:**

The first proband has a de novo balanced translocation t(2;7)(p25.1;q22) identified by karyotyping. Whole genome sequencing showed that the chromosome 7 breakpoint is situated within the SHFM1 locus on chromosome 7q21.3. This separates the *DYNC1I1* exons recently identified as limb enhancers in mouse studies from their target genes, *DLX5* and *DLX6*. In the second family, X-linked recessive inheritance was suspected and exome sequencing was performed to search for a mutation in the affected proband and his uncle. No coding mutation was found within the SHFM2 locus at Xq26 or elsewhere in the exome, but a 106 kb deletion within the SHFM1 locus was detected through copy number analysis. Genome sequencing of the deletion breakpoints showed that the *DLX5* and *DLX6* genes are disomic but the putative *DYNC1I1* exon 15 and 17 enhancers are deleted.

**Conclusions:**

Exome sequencing identified a 106 kb deletion that narrows the SHFM1 critical region from 0.9 to 0.1 Mb and confirms a key role of *DYNC1I1* exonic enhancers in normal limb formation in humans.

Split-hand/split-foot malformation is a congenital limb abnormality characterised by the absence of one or more median rays or digits that results in cone-shaped clefts of hands and/or feet. It is often accompanied by other limb anomalies including monodactyly, syndactyly, and aplasia or hypoplasia of the phalanges, metacarpals, and metatarsals. The malformation can be isolated or syndromic (intellectual disability in 33%, craniofacial malformations in >35%, and deafness in 35%; split-hand/split-foot malformation 1D (SHFM1D), OMIM #220600), and the severity can be variable between patients as well as between different limbs of the same individual. The condition is genetically heterogeneous with six loci defined, including the SHFM1 locus 7q21 (OMIM #183600) and SHFM2 locus Xq26 (OMIM #313350). The 7q21 locus contains the candidate SHFM genes distal-less homeobox 5 and 6 (*DLX5/6*); double-knockout mice exhibit severe skeletal abnormalities and die shortly after birth,^1^ while a homozygous missense mutation in *DLX5* has recently been reported to cause split-hand/foot malformation and hearing loss in a consanguineous family.^2^ No other known *DLX5/6* coding mutations leading to SHFM1 have been reported to date. Several chromosomal abnormalities have been reported in this region that lead to SHFM1; some do not affect the *DLX5/6* genes and are thought to disrupt one or more regulatory elements, or result in a physical separation of such elements from the most likely target genes, *DLX5/6*. The smallest reported deletion is of 880 kb encompassing *SLC25A13* and part of *DYNC1I1*, but leaving *DLX5* and *DLX6* intact.^3^ In the developing limb, both *Dlx5* and *Dlx6* are thought to be regulated by the transcription factor tumour protein p63^4^ and *TP63* mutations cause split-hand/split-foot malformation 4 (SHFM4; OMIM #605289). An enhancer element with a p63 binding site has been reported within the SHFM1 locus 300 kb proximal to *Dlx5/6.*^3^

Recent studies in mouse and zebrafish models identified novel tissue-specific enhancers that correlate with limb, craniofacial and hearing phenotypes observed in individuals with chromosomal rearrangements.^5^ ^6^ Two of these enhancers are located within the coding exons 15 and 17 of *Dync1i1* (*dynein*, *cytoplasmic 1*, *intermediate chain 1*), a gene that is not expressed during limb development. *DYNC1I1* eExon (exonic enhancer) 15 is marked by an enhancer chromatin signature and physically interacts with the *Dlx5/6* promoter regions 900 kb distal to *DYNC1I1,* specifically in the limb.^5^ An additional multi-tissue enhancer was identified in an intron of *Slc25a13* (*solute carrier family 25 member 13*), driving the expression of *Dlx5/6* in otic vesicle, forebrain, branchial arch and limb—a pattern that correlates with some but not all SHFM1 phenotypes observed in humans.^6^

We studied three patients with SHFM from two families. Informed consent was obtained from all participants or their parents. Proband 1 had split-hand/split-foot malformation (figure 1A) affecting three limbs, and sensorineural deafness. Array comparative genomic hybridisation (CGH) analysis indicated normal dosage, but subsequent karyotyping showed a de novo balanced translocation (2;7)(p25.1;q22). Paired-end whole genome sequencing was performed on a single lane of Illumina HiSeq 2000 to map the translocation breakpoints. We obtained average coverage of eight reads per base, and looked for reads where one of the pair mapped at or near the chr2 region, and the other at or near the chr7 region. We identified seven such split pair reads, and a further six reads mapping directly over the translocation breakpoints (see online supplementary figure 1A) which showed that the chromosome 7 breakpoint is within 7q21.3, not 7q22. The precise breakpoints were confirmed by Sanger sequencing of PCR products spanning the breakpoints (see online supplementary figure 1B), and the translocation was defined as t(2;7)(p25.1;q21.3)(oNC_000007.13:g.96229309::CTCTGC::NC_000002.11:g.10585833;NC_000007.13:g.96229314::G::oNC_000002.11:g.10585828). The chromosome 2 breakpoint is located within intron 1 of the ornithine decarboxylase 1 (*ODC1*) gene encoding the rate-limiting enzyme of the polyamine biosynthesis pathway. A polymorphism in this gene has been associated with colon cancer risk,^7^ but there are no reported disease-causing mutations. The chromosome 7 breakpoint is situated within the 7q21.3 SHFM1 locus, in the intergenic region 370 kb upstream of *DLX5/6* genes and 500 kb downstream of the *DYNC1I1* exons 15 and 17 identified as exonic enhancers in mice and zebrafish (figure 2).^5^

**Figure 1:**
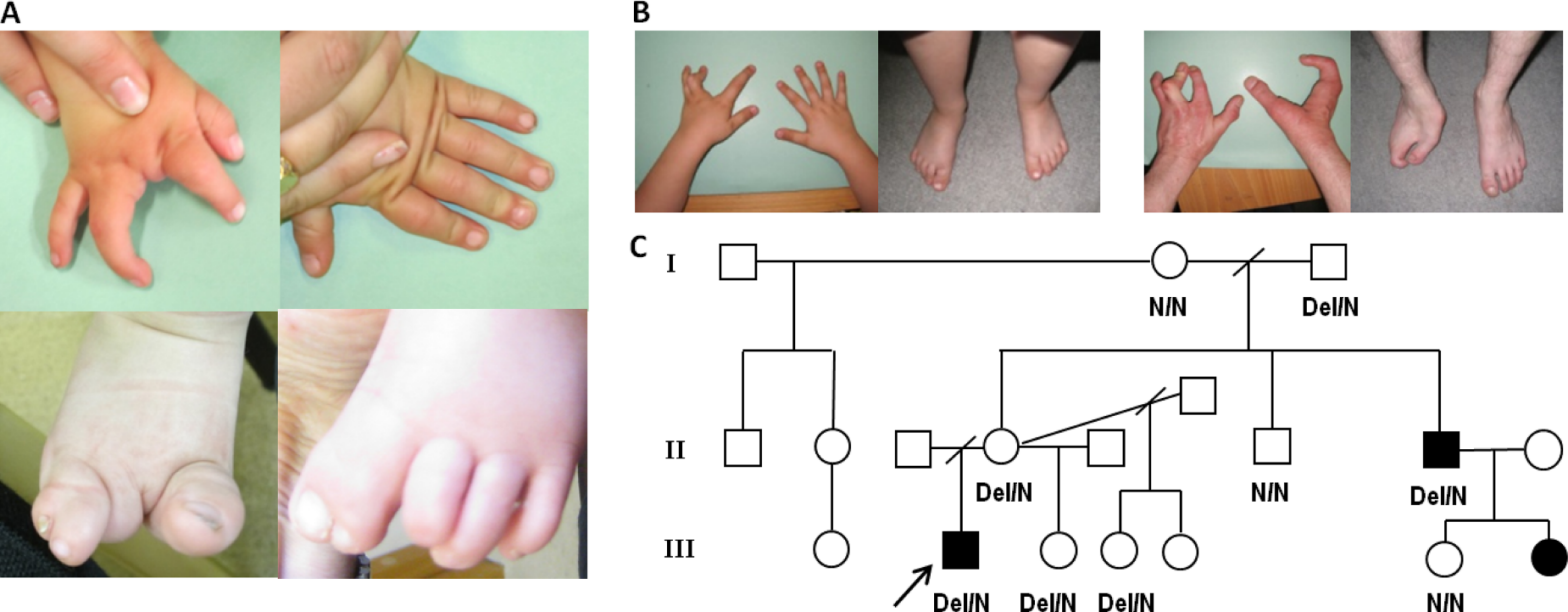
Variable malformation severity and incomplete penetrance in the two split-hand/split-foot malformation 1 (SHFM1) families. (A) Hands and feet of the family 1 patient with a de novo translocation. The right hand has a deficient central ray (upper left image). The left hand is normal (upper right image). The right foot shows deficiency of the second and third rays, partial syndactyly of toes 4–5, and a large great toe (lower left). The left foot shows syndactyly of toes 1–2, and deficiency of the terminal phalanx of the third toe with anonychia (lower right). (B) Hands and feet of the family 2 patients with the deletion. In the proband, both feet and the right hand are normal. In the left hand, the central ray is deficient, and in the ring finger there is camptodactyly at the proximal interphalangeal joint (left image). The proband's maternal uncle is severely affected, with the exception of his left foot, which is normal. His left hand is similar to that of his affected nephew, with deficiency of the central ray, camptodactyly of the ring finger, and some shortening and stiffness of the second (index) finger. In the right hand the second and third rays are absent and there is fusion/syndactyly of rays 4–5 with a single digit. The right foot is also severely affected, with absence of the second and third rays, and fusion of rays 4–5 with a single digit (right image). The uncle's affected daughter (no images) has deficiency of the central ray of the right hand and right foot, with the left hand and left foot unaffected. The right hand closely resembles the left hands of her father and proband. (C) Family 2 pedigree and the results of junction fragment PCR sequencing of both affected (black shapes) and unaffected (white shapes) family members, showing incomplete penetrance of the mutation. The two individuals in whom we performed exome sequencing are the proband (black arrow) and his affected uncle. After the deletion was identified, a third individual (the first female affected in this family) was diagnosed with suspected split-hand/split-foot malformation on routine antenatal ultrasound. She has missing central rays (third digit) of the right hand and right foot only.

In family 2 the proband and his affected uncle have isolated split-hand/split-foot malformation affecting one or three limbs (figure 1B). The uncle has complained of mild hearing difficulty for about 5 years and an audiogram revealed unilateral high tone loss; however, he used to work in a machine tool environment, which may have been the cause. Array CGH analysis using an Agilent 44K oligo array was normal and the mode of inheritance was thought most likely to be X-linked recessive. We performed exome sequencing of both affected individuals to look for shared mutations within the putative SHFM2 region at Xq26.3, but failed to find a likely causal mutation at Xq26 or elsewhere within the exome. We then carried out copy number analysis of the entire chromosome 7 using the method published by Nord *et al*^8^ and identified a single shared ∼100 kb deletion on 7q21, partially encompassing exons 11–18 of the *SLC25A13* gene and exons 14–17 of the *DYNC1I1* gene (see online supplementary figure 1C). Biallelic mutations in *SLC25A13* cause citrin deficiency, an adult- or neonatal-onset metabolic disorder, while homozygous *Slc25a13* knock-out mice show no skeletal defects^9^; hence a heterozygous partial deletion of this gene is unlikely to be associated with SHFM1 phenotype. The *DYNC1I1* gene encodes the intermediate chain 1 subunit of the cytoplasmic dynein motor protein complex, the primary motor protein responsible for retrograde axonal transport in neurons. Since this gene is not expressed in the developing limb a partial gene deletion is not predicted to cause split-hand/foot malformation. However, the deleted *DYNC1I1* exons include the enhancers identified by Birnbaum *et al.*^5^ Visual inspection of the reads indicated that the breakpoints were not located within the exons, so in order to accurately map the deletion we performed paired-end whole genome sequencing of the proband. We looked for read pairs with large (>100 kb) insert sizes within the introns on either side of the last deleted exon, and identified four such read pairs, as well as two reads mapping directly across the breakpoints (see online supplementary figure 1D). Sanger sequencing of a PCR product spanning the breakpoints confirmed a 105 935bp deletion; NC_000007.13:g.95704812_95810747del (see online supplementary figure 1E). The presence of the junction fragment was confirmed in the two affected individuals and also in four non-penetrant unaffected family members (figure 1C). Incomplete penetrance of rearrangements affecting the SHFM1 locus has been reported previously.^10–13^ After the deletion was identified, a third affected individual was diagnosed during routine antenatal scanning (figure 1C). At birth, this baby girl was noted to have a normal left hand and left foot. However, the right hand and right foot were affected to a similar extent, with absence of the central ray and, in the hand, camptodactyly affecting the ring finger, as well as the second (index) finger to a lesser extent.

The 106 kb deletion identified in both affected members of the second family is the smallest deletion identified within the SHFM1 region. It results in haploinsufficiency for most 3′ *DYNC1I1* exons 14–17. This result suggests that *DYNC1I1* exons 15 and 17 act as tissue-specific enhancers of *DLX5/6* expression in humans as well as in mouse and zebrafish. DLX5 and DLX6 are transcription factors essential for epidermal morphogenesis and limb development, and targeted inactivation of the *Dlx5/6* gene pair in mice results in severe limb, craniofacial, and axial skeletal defects.^1^ However, only a single human mutation in *DLX5/6* has been identified to date,^2^ and the reported chromosomal aberrations leading to SHFM1 encompass but the breakpoints do not directly disrupt either of the two genes, suggesting that DLX5/6 *cis*-regulatory elements play a crucial role in human limb development. Interestingly, the deletion in family 2 does not extend to the p63 binding site in the enhancer identified by Kouwenhoven *et al*.^3^

In addition to *DYNC1I1* eExons 15 and 17, several other putative mouse and zebrafish enhancers have been identified within the minimal SHFM1 region that extends between the *DYNC1I1* and *DLX5/6* genes.^6^ The conserved sequences were shown to drive *Dlx5/6* expression in the developing limb, fin, brain, ear, branchial arch (gill and jaw), and genital tissues, and the position of these functional enhancers compared to SHFM1-associated chromosome rearrangements suggests that their disruption might, in some cases, explain the additional clinical phenotypes such as hearing loss and craniofacial defects.^6^ Within intron 14 of *SLC25A13* is an enhancer with activity in the otic vesicle (figure 2) that might explain the sensorineural deafness in proband 1 through the physical separation of this enhancer from the *DLX5/6* genes. Although the deletion in family 2 also includes *SLC25A13* intron 14, only one of the six individuals with the deletion is reported to have mild hearing difficulties, likely to be coincidental, and no other additional clinical features were noted. It is possible that these enhancers either act in a slightly different way in humans compared to mouse and zebrafish, or there may be some redundancy given that several enhancers have been identified for the same tissue, and single enhancers have been observed in multiple tissues.^6^

**Figure 2:**
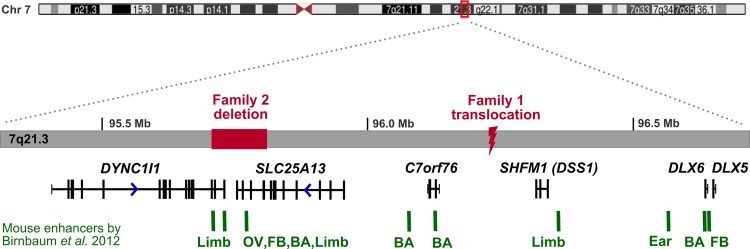
The full SHFM1 locus showing the locations of the two mutations identified in this study relative to the previously published mouse (E11.5) tissue-specific enhancers. The deletion and translocation breakpoints physically separate tissue-specific enhancers from DLX5/6 target genes. BA, Branchial arch, FB, Forebrain; OV, Otic vesicle.

Our whole genome sequencing demonstrates that, even at a relatively low mean coverage (8 reads per base per proband), it is possible to accurately resolve translocation or deletion breakpoints. For proband 1 the genome sequencing was more accurate than metaphase chromosome analysis and resulted in a reassignment of the 7q breakpoint to the neighbouring chromosome band. The application of next generation sequencing for precise characterisation of breakpoints is likely to find clinical utility as it can show whether the coding region of a candidate disease gene is disrupted or, as in this study, identify the spatial relationship between regulatory elements and their target genes.

The use of paired-end whole genome sequencing to map rearrangement breakpoints to the exact nucleotide has previously been described for just seven patients.^14–16^ In all cases the approximate location of the rearrangement, or at least the chromosomes involved, was already known to the analysts, and one of the next challenges is to be able to identify such rearrangements without the need for prior cytogenetic testing. We used the method developed by Nord *et al*^8^ to identify the causative deletion in family 2. A number of different tools have been described for detecting copy number variants (CNVs) from exome data. Some have been successfully applied to discovering CNVs in the general population^17^ ^18^ or in multifactorial disease cohorts,^19^ ^20^ but there are very few examples of novel pathogenic deletions causing monogenic disorders detected through normalised read depth analysis of exome sequence data.^21^

In summary, by using exome sequencing copy number analysis and whole genome sequencing to map deletion and translocation breakpoints, our study demonstrates that exonic enhancers recently discovered through mouse and zebrafish models are also critical for limb development in humans.

## Supplementary Material

Web supplement
