# Supplementary material for: Next generation sequencing of chromosomal rearrangements in patients with split-hand/split-foot malformation provides evidence for DYNC1I1 exonic enhancers of DLX5/6 expression in humans
Source: J Med Genet. 2014 Jan 23;51(4):264–7. doi: 10.1136/jmedgenet-2013-102142 (PMC3963551; doi:10.1136/jmedgenet-2013-102142)
Supplement: Web supplement [file jmedgenet-2013-102142-s1.pdf]

Supplementary Figure 1. Characterisation of translocation (A, B) and deletion (C, D, E) breakpoints.
(A,D) Integrative Genomics Viewer (IGV) plots. The red vertical line in the top panel indicates which portion of the chromosome is displayed, and the blue line below shows the gene covered by the visible part of the chromosome. The third panel is the coverage over the displayed region, on scale 0-20 reads per base. The bottom panel shows all the aligned reads; gray colour indicates that the read sequence matched the reference, while the mismatched bases are shown in yellow, red, green and blue. Reads with 'rainbow' colour blocks suggest that one end of the read does not align at all, and is only partially mapped. Different colours for the entire reads are used to indicate unexpected mapping pattern, such as read pairs mapping to different chromosomes (**A**), or pairs with unexpectedly large or small insert sizes (**D**) resulting from deletions or insertions.
(A) IGV plot of family 1 translocation showing 7 read pairs with one of the pair mapping to chromosome 2 (left panel, blue reads), and the other to chromosome 7 (right panel, red reads). Additionally there are 6 partially mapped reads across the breakpoint. Purple read in the right panel is a read with a missing mate pair.
(B) Translocation breakpoint junction sequence analysis showing chromosome 7, der(2,7), and chromosome 2 sequences, respectively.
(C) Normalised coverage of exome sequence data over the chromosome 7 deletion, showing the exons with reduced coverage in the two affected individuals from family 2 (red and blue lines). Gray lines represent seven unrelated samples sequenced in the same way and used to normalise coverage.
(D) IGV plot of the reads around the deletion breakpoints within the *DYNC1I1* and *SLC25A13* genes in the proband, indicated by 4 read pairs with 106kb insert size (red). The two partially mapped reads resolve the breakpoints at the nucleotide level.
(E) Deletion breakpoint junction sequence analysis showing proximal, recombined and distal sequences, respectively.

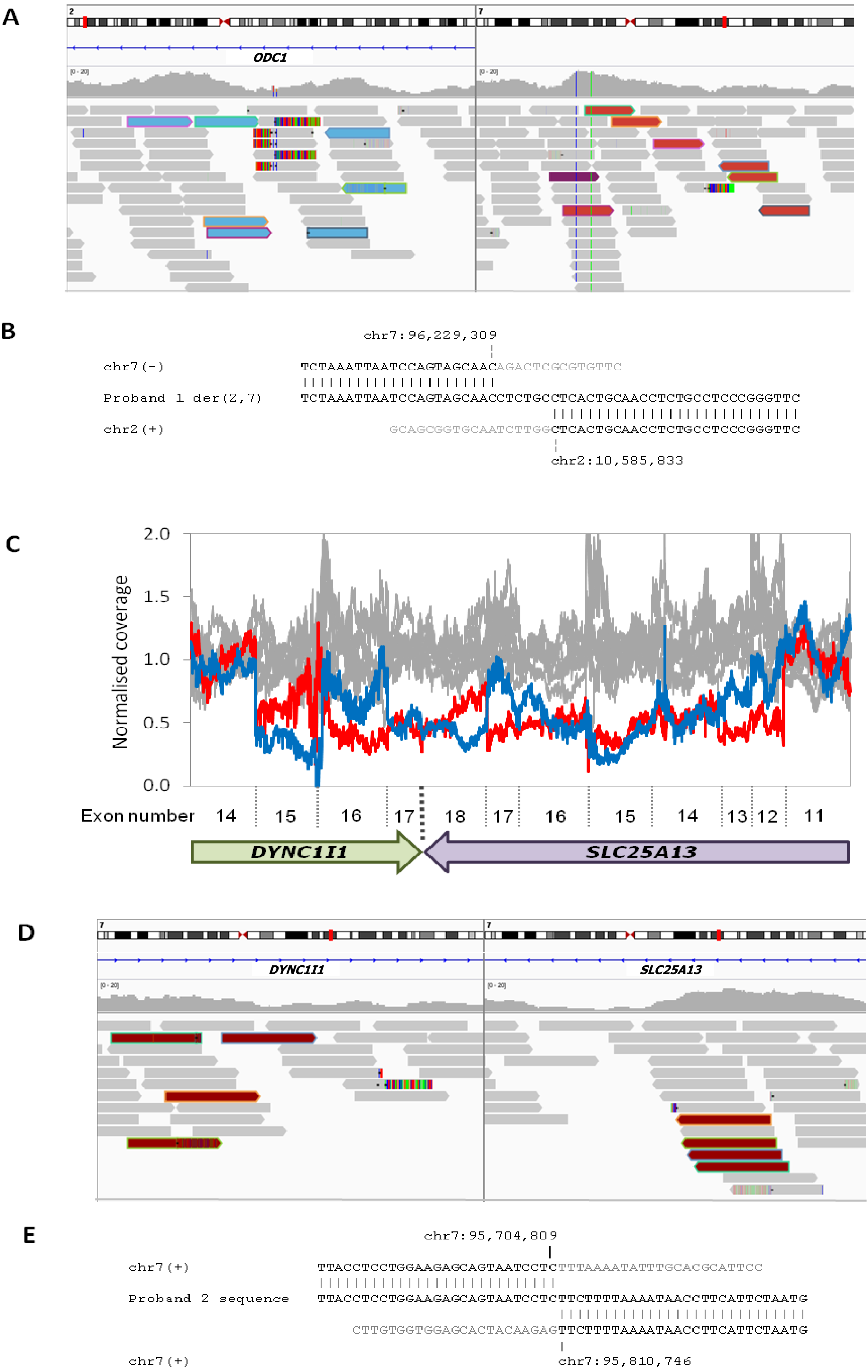
A
2
ODC1
7
[0 - 20]
B
chr7:96,229,309
chr7(-) TCTAAATTAATCCAGTAGCAACAGACTCGCGTGTTC
Proband 1 der(2,7) TCTAAATTAATCCAGTAGCAACCTCTGCCTCACTGCAACCTCTGCCTCCCGGGTTC
chr2(+) GCAGCGGTGCAATCTTGGCTCACTGCAACCTCTGCCTCCCGGGTTC
chr2:10,585,833
C
Normalised coverage
2.0
1.5
1.0
0.5
0.0
Exon number
14 15 16 17 18 17 16 15 14 13 12 11
DYNC1I1
SLC25A13
D
7
DYNC1I1
SLC25A13
[0 - 20]
E
chr7:95,704,809
chr7(+) TTACCTCCTGGAAGAGCAGTAATCCTCTTTAAAATATTTGCACGCATTCC
Proband 2 sequence TTACCTCCTGGAAGAGCAGTAATCCTCTTCTTTTAAAATAACCTTCATTCTAATG
CTTGTGGTGGAGCACTACAAGAGTTCTTTTAAAATAACCTTCATTCTAATG
chr7(+) chr7:95,810,746
